# Beyond Longer Intervals: Advocating for Regular Treatment of Neovascular AMD

**DOI:** 10.3390/jcm14010057

**Published:** 2024-12-26

**Authors:** Alexandra K. Gilligan, David J. Ramsey

**Affiliations:** 1 Department of Ophthalmology and Visual Sciences, Montefiore Medical Center, Bronx, NY 10467, USA; AGilligan@montefiore.org; 2 Division of Ophthalmology, Department of Surgery, UMass Chan—Lahey School of Medicine, Burlington, MA 01805, USA; 3 Department of Ophthalmology, Tufts University School of Medicine, Boston, MA 02111, USA

**Keywords:** retina, neovascular age-related macular degeneration, vascular endothelial growth factor, treat and extend

## Abstract

Personalizing the management of neovascular age-related macular degeneration (nAMD) poses significant challenges for practicing retina specialists and their patients. This commentary addresses some of these complexities, particularly those that arise in the context of an expanding array of intravitreal agents targeting vascular endothelial growth factor (VEGF) and related retinal disease targets. Many of these newer agents approved by the Food and Drug Administration (FDA) for the treatment of nAMD have labeling that indicates that they can provide non-inferior visual outcomes when compared head-to-head with previously available treatments and can be used at significantly extended dosing intervals in some patients. It can be difficult to know if patients should be transitioned to these agents, especially those who are doing well on existing therapies. Although offering extended intervals may be appropriate for some patients with excellent disease control, retina specialists know that undertreatment risks the loss of visual acuity (VA). It can also be challenging for clinicians to interpret the results delivered by clinical trial treatment protocols compared with what is likely to occur in real-world office settings. Many retina specialists use less liberal treatment paradigms than employed in clinical study protocols and consequently many patients experience shorter injection intervals. Since VA is most closely linked to quality of life, it should be prioritized compared with other endpoints. The authors advocate for maintaining consistent treatment schedules dictated by disease control instead of switching therapies even in the presence of small amounts of macular fluid that may occur with longer injection intervals.

## 1. Introduction

Age-related macular degeneration (AMD) is a leading cause of visual impairment and blindness among older adults and effects more than 200 million people worldwide [1]. As the population ages and life expectancy increases, the number of individuals reaching the advanced stages of the disease expands, making AMD an increasingly important public health issue [2]. The prevalence of early-stage AMD in the United States was 12.7% in 2019, ranging from 2.0% among Americans aged between 40 and 44 inclusively to 35.2% among Americans aged 85 years or older [3].

**Figure 1:**
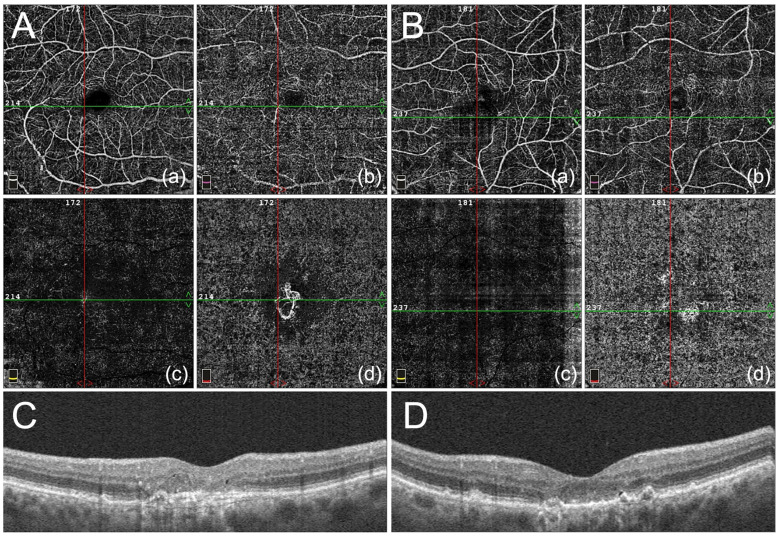
Regular treatment of nAMD can deliver excellent, long-term visual outcomes. Shown is an example patient who helped inspire this comprehensive review of the importance of regular treatment for nAMD. She is an 89-year-old woman with bilateral nAMD on monthly treatment with off-label bevacizumab (1.25 mg; Avastin^®^; Genentech Inc., San Francisco, CA, USA). The patient’s VA was 20/200 OD (right eye) and 20/20 (−2) OS (left eye). Being functionally monocular, she elected to continue monthly bilateral treatment to preserve her current VA. Optical coherence tomography (OCT) angiography of the right (**A**) and left (**B**) eye show depth-resolved images of the superficial (**a**) and deep capillary plexuses (**b**) of the retinal microvasculature, as well as the avascular outer retina (**c**) and choriocapillaris layers (**d**), which demonstrate choroidal neovascularization in the macula of both the right eye (which eventually stopped being treated) and left eye (still 20/20 on treatment administered every six to eight weeks, more than five years later). Images were segmented by using the built-in software (6 × 6 mm, RTvue XR Avanti, AngioVue software, version 2017.1.0.149, Optovue, Inc., Fremont, CA, USA). Vertical spectral-domain macular OCT scans are shown through the fovea of the patient’s right (**C**) and left (**D**) eyes, respectively. Beneath the retina, there is drusen with low internal reflectivity. In the right eye, there is loss of signal attributable to the ellipsoid zone (EZ) band with thinning of the outer retinal layers and early hypertransmission corresponding to loss of the retinal pigment epithelium/Bruch’s membrane complex.

Agents targeting vascular endothelial growth factor (VEGF) have revolutionized the management of neovascular age-related macular degeneration (nAMD). Regular injections of anti-VEGF agents can suppress disease activity and preserve vision even when macular neovascularization (MNV) is present (Figure 1). In contrast, there are no equally effective treatments to maintain vision in patients with advanced non-exudative AMD. However, recent clinical trials of agents inhibiting the complement system have demonstrated success in slowing anatomical progression of geographic atrophy (GA), a late-stage version of dry AMD that leads to severe vision loss [4]. These findings provide evidence that these treatments may help preserve visual function [4].

It is imperative for clinicians to understand how to individualize the care for their patients burdened by nAMD. A recent study showed that, concerningly, 11.6% of patients with newly diagnosed nAMD were lost to follow-up over a four-year period [5]. This gap in care delivery illustrates an opportunity to avoid preventable vision loss. Furthermore, as many as 14.0% of patients actively treated for nAMD admit to missing a scheduled injection visit [6]. In our practice, 7.5% of patients with nAMD became lost to follow up for longer than one year [7]. Barriers to adherence with therapy include absence of symptoms, financial considerations related to examination costs, extended clinic wait times, travel difficulties, forgetting about appointments, scheduling difficulties, dissatisfaction with the benefits of treatment, factors associated with older age, and poorer vision [7,8,9]. Personalizing approaches to treatment within an evolving landscape of medications is important for optimal care of patients and those who support them because many have competing health and life priorities. Amid these factors, the optimal treatment strategy, medication intervals, and threshold for switching agents remain areas of debate among ophthalmologists.

Intravitreal injection of anti-VEGF agents is the mainstay of nAMD treatment (Table 1). The results from large, multicenter, blinded, randomized control trials (RCTs) constitute the benchmark for visual and anatomical outcomes obtainable from the optimal management of nAMD. The modern anti-VEGF era was ushered in with the MARINA study (Minimally Classic/Occult Trial of the Anti-VEGF Antibody Ranibizumab in the Treatment of Neovascular AMD), a multicenter, 2-year, prospective, placebo-controlled trial in which intravitreal ranibizumab (Lucentis^®^, Genentech, Roche Holding AG, South San Francisco, CA, USA) demonstrated a significant VA benefit for patients with MNV secondary to nAMD in comparison to sham injections [10]. A few years later, aflibercept 2 mg (Eylea^®^, Regeneron, Tarrytown, NY, USA) was demonstrated to have non-inferior gains in VA compared to ranibizumab in the Vascular Endothelial Growth Factor Trap-Eye: Investigation of Efficacy and Safety in Wet Age-Related Macular Degeneration (VIEW) 1 and VIEW 2 studies, including at intervals extended up to eight weeks [11]. More recently, faricimab-svoa (Vabysmo^®^, Genentech, South San Francisco, CA, USA) similarly demonstrated non-inferior VA claims compared to aflibercept 2 mg restricted to 8-week dosing in the TENAYA and LUCERNE phase 3 trials [12] (summarized in Table 2).

Within the context of these trials, longer intervals of up to 16 weeks were allowed for patients treated with faricimab, and the results demonstrated superior retinal drying even at these extended intervals for up to 63.1% patients in the twin studies [13]. This outcome has been attributed to the bispecific design of faricimab, which also targets angiopoietin-2 (Ang-2) [12,13]. There are, however, no controlled trials demonstrating that the portion of the molecule targeting Ang-2 is responsible for any of the clinical effects despite compelling preclinical evidence [14]. In the TENAYA and LUCERNE trials, faricimab resulted in faster and greater improvement in retinal anatomy compared to aflibercept, with 60% of patients achieving absence of retinal fluid by week 4 and 77% by week 12. Though there was a rapid reduction in retinal fluid and controlled nAMD activity, visual improvement was similar between the two treatments, which highlights the complex relationship between anatomical changes and visual outcomes. Furthermore, faricimab-treated patients in the TENAYA and LUCERNE trials showed faster retinal fluid reduction and greater absence of fluid compared with aflibercept 2 mg, potentially permitting an earlier extension of dosing intervals, resulting in fewer injections to maintain disease control [15]. 

Adding to the physician’s repertoire, a high molar concentration version of aflibercept (8 mg Eylea HD^®^, Regeneron Pharmaceuticals, Tarrytown, NY, USA) has recently been shown to have non-inferior visual outcomes at up to 16 weeks after three initial monthly doses in 77% of patients randomized to this dose and regimen [16]. 

Finally, off-label bevacizumab (1.25 mg; Avastin^®^; Genentech Inc., San Francisco, CA, USA) was shown to provide non-inferior gains in VA compared with ranibizumab in the Comparison of Age-related Macular Degeneration Treatments Trials (CATT) trial when used at monthly intervals [17,18].

Another agent that bears mention is brolucizumab (Beovu^®^, Novartis, Basel, Switzerland), which was FDA-approved for nAMD in 2019. However, it is very infrequently administered within the United States in part because it has been associated with intraocular inflammation [19,20], for which it has a Black Box warning from the FDA in 2021. Thus, the authors of this commentary do not discuss the use of that agent.

**Table 1:** Ranibizumab, aflibercept, brolucizumab, faricimab and bevacizumab (off-label) are agents administered by intravitreal injection used by retina specialists to treat nAMD [10,11,12,13,18,20,21].

	Ranibizumab	Aflibercept	Brolucizumab	Faricimab	Bevacizumab
**Mechanism**	Humanized antibody fragment region that targets vascular endothelial growth factor A (VEGF-A)	“Decoy” receptor for VEGF and placenta growth factor that prevents them each from binding to their respective receptors	Humanized single chain antibody fragment that binds to VEGF-A	Humanized IgG1 and 3 human IgG1λ antibody that block both VEGF-A and Ang-2	Full-length monoclonal antibody that targets VEGF-A
**Dose**	0.5 mg (0.05 mL)	2 mg (0.05 mL) or 8 mg (0.07 mL)	6 mg (0.05 mL)	6 mg (0.05 mL)	1.25 mg (0.05 mL)
**Frequency of Administration**	Monthly (approximately every 28 days) Alternatively, although not as effective, patients may be treated with 3 or 4 monthly doses followed by less frequent dosing.	Initial phase: 2 mg (or 8mg) every 4 weeks (monthly) for the first 3 months. Maintenance Phase: 2 mg every 8 weeks (bimonthly) thereafter (or 8 mg every 8 to 16 weeks (±1 week).	Initial Phase: Every 4 weeks (monthly) for the first 3 months. Maintenance Phase: every 8 to 12 weeks	Initial Phase: every 4 weeks (approximately every 28 days) for the first four doses. Maintenance Phase: Subsequent doses administered every 8 to 16 weeks, based on individual patient needs guided by OCT and VA evaluations.	Typically given initially monthly, followed by OCT and VA evaluations to guide further treatment

With the burgeoning array of treatment options for nAMD, physicians find themselves navigating an increasingly complex landscape. Amidst this abundance, they bear the crucial responsibility of interpreting and adapting diverse clinical trial data to guide their clinical practice. This can be quite complex for physicians because retreatment criteria are often significantly more liberal in studies compared with those typically employed by retina specialists in clinical practice. For example, TENAYA and LUCERNE trials extended in intervals of four weeks and tolerated up to a 50-µm increase in central subfield thickness (CST) or a decrease of up to five letters in best corrected visual acuity (BCVA) compared with the average of the two prior visits, provided there was no macular hemorrhage observed [13]. In stark contrast, many retina specialists utilizing treat-and-extend (TAE) in clinical practice seek to maintain a dry macula and often extend in increments of as little as one to two weeks with the goal of maintaining VA [9,22]. This more conservative approach to TAE has also been incorporated into a number of prospective clinical trials [23,24].

With the burgeoning array of options and the rapid accumulation and analysis of data, physicians find themselves navigating a landscape rich with possibilities. Yet, amidst this abundance, they are tasked with the crucial responsibility of adeptly interpreting such information and discerning the most effective means of integrating it into their clinical endeavors. This commentary addresses many of these complexities and advocates for maintaining consistent treatment schedules for nAMD aim at disease control because undertreatment risks the loss of visual acuity (VA) and vision-associated quality of life.

## 2. Materials and Methods

In this study, the authors made a comprehensive review of the existing literature and published data from multiple landmark trials and reputable sources, including peer-reviewed journals and clinical trials. The databases Web of Science and PubMed were queried between January 2004 to November 2024 by using the combination of the keywords “neovascular age-related macular degeneration”, “visual acuity”, “treat and extend” (41 results) and “neovascular age-related macular degeneration” and “treat and extend” with specific drug names (ranibizumab (133 results), bevacizumab (45 results), faricimab (33 results), and aflibercept (126 results)). We excluded duplicate publications, articles not in English, and unrelated content lacking discussion of drug therapy or treatment outcomes. We focused on trials published within the last two decades to ensure the data’s relevance and applicability to current clinical practice. To provide robust evidence, we gave priority to RCTs, meta-analyses, and cohort. In total, 85 full articles were evaluated, and 56 are included and cited in this paper. Data were systematically extracted from the selected studies, including but not limited to participant demographics, intervention details, outcome measures, and statistical results. Finally, the synthesized findings were critically analyzed, and unified conclusions were drawn based on the collective evidence. Recommendations for clinical practice and future research directions were also formulated based on identified gaps in the literature.

## 3. Results and Discussion

The analysis presented seeks to establish the importance of maintaining treatment at regular intervals rather than immediately transitioning therapies because of small amounts of macular fluid. Some eyes may respond in time if given regular treatments. In other cases, treatment at relatively less extended intervals may provide better disease control, especially when patients experience an unanticipated delay in returning for eyecare. Longer intervals may also magnify the time of treatment interruption when a patient returns for their next interval treatment, thereby risking vision loss.

The CATT trial demonstrated that the administration of bevacizumab and ranibizumab injections monthly was superior to a pro re nata (PRN) approach where treatment decisions were guided by optical coherence tomography (OCT) findings. At both the one-year and two-year endpoints, patients treated monthly had both better VA outcomes and more effective resolution of fluid [18,21]. Most patients and their physicians value the most VA as the endpoint of greatest concern. Evidence indicates that eyes without having or with only extrafoveal fluid achieve and maintain better VA compared with those with fluid in the fovea (achieving 70.9 and 68.7 Early Treatment Diabetic Retinopathy Study [ETDRS] letters versus 62.3 ETDRS letters, respectively, *p* < 0.001) [25]. On this basis, the prevailing paradigm guiding the visit-to-visit treatment decisions by most retinal specialists in clinical settings emphasizes minimizing fluid presence. It is important to note, however, that the presence of fluid is not inevitably associated with poor visual outcomes. Many patients can achieve improved VA outcomes with monthly injections despite a lack of fluid resolution at the end of one year, and sometimes improvement occurs after as few as three initial monthly injections.

Nevertheless, these anatomical findings are rooted in analyses that do not consider the critical factor of fluid type and location. Subsequent revelations from the same trials (summarized in Table 2) demonstrated that lingering intraretinal fluid (particularly in the foveal region) had a substantial detrimental impact on VA (68.7 ETDRS letters for non-foveal intraretinal fluid (IRF), and 62.3 ETDRS letters for IRF (*p* < 0.001)) [25]. By contrast, non-foveal subretinal fluid (SRF) did not produce a comparable reduction in vision (71.7 ETDRS letters for non-foveal SRF). As such, it would appear prudent to treat patients who have IRF more aggressively compared with patients having SRF, especially when that fluid involves the fovea. In our experience, as patients achieve better disease control, the benefit of more frequent treatments appears to diminish, suggesting that less aggressive management of minimal amounts of fluid may be better tolerated later in the course of disease [9]. Gianniou et al. [25] have demonstrated that the majority of patients with treatment-resistant fluid after one year on ranibizumab maintained VA, with nearly a third achieving an increase of ≥3 lines of VA. However, because complete fluid resolution correlates with greater improvements in vision [21], minimizing fluid is a logical goal.

**Table 2:** Outcome data on visual acuity (VA) based on fluid status (type, persistence) from multicenter, blinded, randomized control study data for various anti-VEGF agents.

VA Based on Fluid Presence									Study Design
**Fluid Presence** **Based on Location**	Drug Regimen	Week 0	Week 4	Week 12	Week 24	Year 1	Year 2	Year 3	
**No fluid** VA in number of letters (number patients in group) [21]	Monthly, PRN ranibizumab and bevacizumab ^‡^ (pooled, *n* = 1140)	- (0)	66 (251)	68 (297)	69 (247)	70 (297)			Multicenter, blinded, randomized control study
**Non-foveal fluid** VA in number of letters (number patients in group) [21]		62 (193)	64 (264)	68 (246)	68 (291)	68 (320)			
**Foveal fluid** VA in number of letters (number patients in group) [21]		60 (947)	64 (582)	65 (483)	66 (463)	68 (414)			
**VA based on Type of Fluid**									
**No IRF** Adjusted mean VA score (SE) [21]	Monthly, PRN ranibizumab and bevacizumab (pooled, *n* = 1004)					70.9 (0.68)			Multicenter, blinded, randomized control study
**IRF but non-foveal** Adjusted mean VA score (SE) [21]						68.7 (0.88)			
**Foveal IRF** Adjusted mean VA score (SE) [21]						62.3 (1.27)			
**No SRF** Adjusted mean VA score (SE) [21]						67.8 (0.61)			
**SRF but non-foveal** Adjusted mean VA score (SE) [21]						71.7 (1.29)			
**Foveal SRF** Adjusted mean VA score (SE) [21]						70.4 (1.29)			
**Change in VA despite 12 months of Persistent Fluid**									
**Mean change of VA** (despite 35.5% of patients resolving) Letters (SD) [25]	Monthly ranibizumab **(*n =* 76)**	Fluid (persistent for 12 months)				9.0 (13.5)	7.9 (15.4)	7.9 (14.8)	Single-center retrospective chart review
**Improved VA** Gained >15 Letters (number of patients/ patients total) [25]						30.3% (23/76)	36.8% (21/57)	26.7% (8/30)	
**Maintained VA** Lost <15 Letters (number of patients/ patients total) [25]						97.4% (74/76)	94.7% (54/57)	90% (27/30)	

^‡^ Monthly treatments were specified as 0.5 mg (0.05 mL) of ranibizumab or 1.25 mg (0.05 mL) of bevacizumab, whereas PRN eyes were evaluated approximately every 28 days and treated with the appropriate drug when there was fluid present on OCT or other signs of neovascularization.

Despite the overall efficacy of anti-VEGF agents, some patients experience treatment-resistant nAMD. This is defined as persistent or worsening macular fluid despite regular anti-VEGF therapy. Sometimes, this is apparent after as few as three initial intravitreal injections [26,27]. More often, patients previously responsive to a specific agent, frequently at extended intervals, develop recurrent fluid necessitating shortened treatment intervals to achieve fluid-free macula, or patients fail to achieve disease control altogether [9]. If reducing the treatment interval proves unsatisfactory because of ongoing nAMD activity, e.g., at 4-week or “monthly” injection intervals, a switch in medications is often initiated. In other situations, an inability to extend intervals beyond a longer interval may also prompt a change in medication in an effort to alleviate the burdens of treatment. In these patients, a switch from a medication used every 4 to 8 weeks to one used at a maximum of every 16 weeks could result in between two and three fewer injections a year [28]. However, there remains the question of when to switch agents to maximize VA outcomes. No studies have established the most ideal time for switching agents when a patient does not respond to treatment. Some physicians limit injections to as few as two to three injections before initiating a switch, while others wait until after four to six injections before changing medications [9,29].

In a retrospective study of patients resistant to treatment resistant nAMD by Hamid et al. [29], it was demonstrated that transitioning to intravitreal aflibercept 2 mg therapy from bevacizumab or ranibizumab injections for treatment-resistant nAMD produced notable short-term visual improvement and a sustained reduction in central macular thickness (CMT) over one year of follow-up. Importantly, patients required less frequent intravitreal injections to achieve these visual benefits (mean of 7.36 ± 1.85 bevacizumab or ranibizumab injections versus 6.47 ± 2.45 aflibercept 2 mg injections (*p* = 0.001). Moreover, the mean change in BCVA from baseline was statistically significant, registering at logMAR 0.05 ± 0.13 (*p* = 0.01) at one month but was similar by one year (logMAR 0.02 ± 0.28, *p* = 1.00) [29]. However, these findings call for careful consideration, given the inherent limitations associated with a retrospective study design. A retrospective, cross-sectional study recently completed demonstrated that switching from bevacizumab or ranibizumab to aflibercept 2 mg for treatment-refractory nAMD did not reduce the treatment index, defined as the rate of injections relative to the maximum number of injections based on medication labeling, or impact adherence, with stable VA despite short-term anatomical changes [9]. Notably, in the VIEW 1 and 2 studies, aflibercept 2 mg was associated with a higher percentage of patients achieving a fluid-free macula, but that result was not associated with better VA in these double-blind clinical trials [11].

The decision to switch is often made more difficult because retina specialists have suboptimal data regarding their patient’s VA. Many retina clinics rely on presenting Snellen acuity instead of measuring BCVA by using a modified ETDRS protocol which is commonly used in clinical trials. This may result in an underestimate of VA or its change (see below) [30]. Taking into account structural biomarkers, though less certain in terms of their implications for long-term VA outcomes, is more objective because these measurements are derived from imaging data. There are also concerns about publication bias, given the tendency for both researchers and journal editors to favor publishing positive results [31]. The Aflibercept as a second line therapy for neovascular age related macular degeneration in Israel (ASLI) study demonstrated that patients who had received three to five previous bevacizumab injections before being switched to aflibercept 2 mg achieved an improved BCVA (60.4 ± 11.2 ETDRS letters versus 62.8 ± 18.7 at week 28, *p* = 0.04) [32]. This difference of less than three ETDRS letters corresponds to less than one line of vision, a difference that though favored by physicians may not be noticeable to patients.

A randomized clinical trial that compares one group of patients on monotherapy receiving one of the available anti-VEGF agents, with a second group in which patients were allowed to switch from one anti-VEGF agent to another based on pre-defined inadequate treatment response criteria would allow for such a comparison of outcomes. Such data could also be used to understand the incremental cost-effectiveness ratio (ICER) and cost per quality-adjusted life-year (QALY) for patients with nAMD. When evaluating the balance between treatment effectiveness and cost, studies often focus on the price of the drug and the physician’s time. However, a recent review of literature and scenario analysis involving an expert panel of ten UK ophthalmologists demonstrated that this technique oversimplifies and underestimates the “cost” analysis portion when discussing nAMD [33]. It fails to account for many expenses such as extended clinic hours and emergency visits. Identifying and allowing for such costs are key for making well-informed treatment decisions and properly assessing cost/benefit analyses for nAMD [1]. In a bid to manage expenses and standardize patient outcomes, health insurance companies are increasingly implementing step therapy [34]. This requires the use of a preferred agent before one or more secondary agents are utilized. Given that existing VEGF agents are, for the most part, non-inferior on the surface, these requirements may seem reasonable. However, these mandates make it all the more important for knowledge to be acquired to comprehend the long-term ramifications of switching therapy.

Unfortunately, primary data from phase III multicenter, blinded, randomized control studies are not easily accessible for all FDA-approved treatments for nAMD. Access to de-identified individual patient-level data and supporting documentation is being made available through data sharing platforms; however, the ease and speed with which these data can be accessed vary [12]. More importantly, multicenter, blinded, randomized control studies of a TAE paradigm need to be investigated more rigorously, especially given that most retina specialists favor this approach in real-world settings (66.7% prefer this strategy for their nAMD patients) [35]. Furthermore, the reason for the mean discontinuation rate of 10.8% for patients enrolled in clinical trials of anti-VEGF agents for treatment of nAMD is unknown [36]. The burdens associated with frequent treatments, including travel, the time spent at appointments, and other direct and indirect costs, influence the decision to adopt a TAE approach [37]. There may also be considerations related to clinic capacity and service costs that influence practices to seek to extend the interval between patient visits [33].

In clinical trial settings, the Trial of Treat-and-Extend versus Monthly Dosing for Neovascular Age-Related Macular Degeneration (TREX-AMD), TReat and extEND (TREND), and Canadian Treat-and-Extend Analysis Trial (CANTREAT) trials have demonstrated the non-inferiority of ranibizumab on a TAE schedule as compared to monthly injections in patients requiring up to one year of treatment [24,38,39]. Other studies have demonstrated that the TAE regimen is superior to PRN and comparable to monthly injections in the short term. As an example, analysis of four ranibizumab studies demonstrated visual gains were higher in the TAE compared to the PRN group (+6.18 letters, 95% CI: 3.28–9.08) [37]. Patients treated with TAE required a mean of 1.44 more injections over a period of 12 months compared to those on the PRN regimen, though with fewer visits since they did not need to be evaluated as often [37,40,41]. Interestingly, although retinal specialists who were polled preferred a TAE paradigm in theory, they were more than 8.5 times more likely to choose monthly dosing if it was their own eye requiring treatment (*p* = 0.006) [42], which is even more conservative than clinical trials may suggest they need to be.

Notably, Javidi et al. [43] looked at longer timepoints and demonstrated in a retrospective cohort study, outlined in “Long Term Visual Outcomes for a Treat-and-Extend Antivascular Endothelial Growth Factor Regimen in Eyes with Neovascular Age-Related Macular Degeneration: Up to Seven-Year Follow-Up” that 180 patients on a TAE regimens with patients on three medications (aflibercept 2 mg, bevacizumab, and ranibizumab) had long-term (defined as greater than six year) stability with an average of six injections per year (higher in the first year but steadily declining afterwards). In the patients that were followed for at least five years, there was a trend toward improvement in VA during the first year (although this did not reach statistical significance), followed by maintenance of the baseline VA for five years. The study by Hamid et al. [29] also reported a reduction of around one fewer injection per year after one year of treatment for those on aflibercept 2 mg compared to bevacizumab and/or ranibizumab, which could be meaningful over a longer term, though additional studies would be needed to reach this conclusion. Finally, Berg et al. [44] showed in a multicenter, randomized trial that bevacizumab and ranibizumab had statistically equivalent effects on maintaining VA following a TAE regimen at one year and that these VA outcomes were comparable to the results of clinical trials that used monthly treatment protocols [35]. When considering patient burdens and the fear or anxiety surrounding long-term need for injections, minimizing these by using a TAE protocol may help to alleviate such issues.

An additional question that emerges pertains to the discernment, if any, of outcomes stemming from periodic monthly interventions as delineated within previously discussed clinical trials, when juxtaposed with the pragmatic implementation of a TAE regimen within an authentic clinical “real-world” setting. As previously deliberated, the TAE paradigm has exhibited its efficacy in clinical trials, manifesting similar visual outcomes to those of monthly injections [17,18]. Nevertheless, it is noteworthy that an assortment of inquiries has delved into retrospective analyses, revealing that the practical deployment of TAE protocols in real-world scenarios has yielded outcomes that fall short of rigorous “clinical trial” standards. Skelly et al. [45] retrospectively analyzed TAE patterns in Australia, where they demonstrated that fewer than 50% of the treatment-naive patients with nAMD were able to sustain stable 12-week treatment intervals, with the majority requiring injections more than every 8 weeks. The mean change in VA from baseline peaked at +6.3 letters at six months but regressed by +3.2 letters by 2 years.

Another challenge in comparing studies and determining the reliability of these data centers around VA measurements. Throughout history, the Snellen chart has been widely used to assess VA, despite notable shortcomings in its precision [30]. In order to minimize inconsistencies and improve the accuracy of vision assessment, more recent charts have been suggested. Among these, the ETDRS chart, derived from the Bailey–Lovie chart and evaluated using the ETDRS protocol, stands as the present benchmark for vision evaluation in clinical trials due to its standardized administration and scoring methods. Kalpana and her colleagues [46] noted a distinct trend where individuals with diminished visual capacity displayed a significantly greater likelihood of achieving improved BCVA measurements when utilizing the ETDRS chart as compared with routine acuities measured by means of Snellen charts. Therefore, many retrospective studies that present Snellen acuity add a further layer of difficulty when comparing non-randomized data that are available to randomized control trials because it is both less precise and less accurate compared with ETDRS-based VAs.

Without multicenter, blinded, randomized control studies, we cannot say with certainty but would surmise that these regimens are likely non-inferior or only marginally inferior to scheduled regimens. When weighed against the burdens of treatment experienced by the patient (most often cited by patients are side effects from injections, concern or fear about injections, and the frequency of visits [6]), TAE regimens may nonetheless be reasonable. However, judging the extent of these differences, if any, is limited by the use of presenting VA in the real-world clinical context where these pharmaceutical interventions are deployed and most often available for study. While acknowledging the existence of these discernible disparities, it is plausible that their magnitude is sufficiently modest to obviate significant concern. This is particularly pertinent when juxtaposed with the consideration of regular intervals for comparison. Considering the efficacy of currently available treatments, most retina specialists feel it is advisable to adopt a gradual and measured approach to extension (at intervals of two weeks) for individual patients who demonstrate stable or fluid-free OCT images [47]. In clinical trials, extension is often conducted in 4-week intervals to permit the necessary synchronization of data collection [11,12].

Few clinical trials have reported OCT-derived structural markers of disease, such as type and location of fluid, which may serve as biomarkers predictive of visual outcomes [48]. Furthermore, without data clarifying the type of breakthrough fluid that occurs when agents are utilized at variable and longer intervals, it is difficult to know how to interpret these paradigms or to determine whether they truly are the equivalent of results that could be achieved with more regular treatment paradigms that might allow for even tighter control of disease activity. This gap in the data impedes physicians and patients from making informed decisions collaboratively based on what could be valuable and objective information related to disease activity.

Available evidence also suggests that less-than-optimal visual outcomes occur when patients are undertreated relative to monthly injections used in clinical trial protocols [18,49]. Another limitation is that RCTs have strict inclusion/exclusion criteria that do not apply to patients in clinical practice. In real-world settings, patients are often older, have greater numbers of comorbidities, and present at significantly more varied disease stages [50]. Patients face barriers to consistent follow-up and frequent monitoring in clinical practice, which may contribute to undertreatment and poorer outcomes with anti-VEGF therapies [9]. There is a tension in real-world practice between offering patients proven, clinical trial-based treatment regimens, or utilizing alternative treatment regimens that require fewer visits and anti-VEGF injections, (e.g., TAE protocols), which reduce for patients and those involved in their care the burden of frequent visits for injections. Importantly, as outlined in Table 3, the percentage of eyes completely fluid-free was always higher in the group receiving monthly injections compared with those receiving PRN injections, regardless of the agent (when looking at ranibizumab, bevacizumab, and aflibercept 2 mg) [3,17,18,51].

It is also important to consider changing agents when a patient is not responding appropriately. Many studies have looked at fluid status in eyes from patients who switched from one anti-VEGF agent to another because of treatment refractory disease. Patients who switched from ranibizumab to aflibercept 2 mg had significantly less IRF, identified in 60% of eyes versus 71% for those that did not switch, though with a non-significant change in VA or CRT [52]. Patients switched from bevacizumab to ranibizumab had a significant improvement in VA, though the specific numbers were not provided, and a decrease in macular thickness by 66 µm from baseline (also significant) [53]. In comparison, those undergoing the reverse switch, from ranibizumab to bevacizumab, did not have an improvement in VA or central retinal thickness [53]. In yet another study, switching from aflibercept 2 mg to ranibizumab did not yield any significant change in VA [54]. Similar retrospective studies are now being undertaken with newer agents, like faricimab. When patients who had undergone at least fifteen prior injections with at least two different anti-VEGF agents were switched to faricimab, 47% of eyes with previously persistent IRF and SRF became fluid-free [55]. nAMD patients receiving bevacizumab and/or aflibercept injections that were switched to faricimab had a decrease of 34.0 µm in their central subfield thickness at their most recent follow-up appointment, supporting the conclusion that this medication is a stronger drying agent. However, this study, as well as results from our own academic center [22], found no significant change in VA among such patients. Finally, a pilot study of treatment-naive eyes with nAMD that underwent three consecutive monthly injections with either aflibercept 2 mg or bevacizumab, followed by a TAE protocol, in which the decision to extend the interval between treatments was based on VA, clinical exam, and the continued absence of fluid on OCT found that a great number of eyes were able to be weaned off treatment when initially treated with aflibercept 2 mg compared with bevacizumab (43% versus 15%, *p* < 0.001) [56]. Future studies are needed to determine the safety and appropriateness of monitoring patients on such PRN regimens.

## 4. Conclusions

Determining if patients with well-controlled nAMD on their present regimen could benefit from a switch to newer agents whose labels indicate that they can be used at longer intervals is unknown. In some cases, patients who are able to achieve remission can even suspend anti-VEGF therapy without immediate recurrence of disease, though the durability in all such cases is unknown. Finally, for patients who are not optimally controlled with ongoing disease activity, switching agents should be considered because the balance of prevailing evidence points to at least short-term visual gains accompanied by anatomical improvements. In such cases, it is important to take account of the type, location, and extent of fluid because not all fluid has the same implications for VA. However, properly designed clinical trials to understand better the dynamic interplay between switch in agent and long-term visual outcomes are needed. Until then the paradigms used by retina specialists to determine when it is best to switch agents will continue to be based on expert judgment guided by the individual needs of each patient.

## Figures and Tables

**Table 3:** Outcome data on visual acuity (VA) and fluid status from multicenter, blinded, randomized control study data for outcomes with agents targeting VEGF.

	Drug Regimen	Year 1	Year 2	Study Design	
**Increased VA** Gained >15 letters % (number of patients /total patients)	Ranibizumab q4 weeks [17,18].	34.2 (97/284)	32.8 (44/134)	Multicenter, blinded, randomized control study	
	Bevacizumab q4 weeks [17,18]	31.3 (83/265)	31.8 (41/129)		
	Aflibercept 2 mg q4 weeks [11,51]	37.5 (114/285)	28.1 (168/597)		
	Aflibercept 2 mg q8 weeks [11,51]	30.6 (92/265)	33.4 (203/607)		
**Maintained VA** Lost <15 letters % (number of patients /total patients)	Ranibizumab monthly [17,18]	94.4 (268/284)	93.3 (125/134)	Multicenter, blinded, randomized control study	
	Bevacizumab monthly [17,18]	94.0 (249/265)	92.2 (119/129)		
	Aflibercept 2 mg q4 weeks [11,51]	95.1 (271/285)	91.5 (546/597)		
	Aflibercept 2 mg q8 weeks [11,51]	95.1 (252/265)	92.4 (561/607)		
**Fluid Absence** % (number of patients /total patients)	Ranibizumab monthly [17,18]	43.7 (124/284)	45.5 (61/134)	Multicenter, blinded, randomized control study	
	Ranibizumab PRN [17,18]	23.9 (68/285)	22.3 (59/264)		
	Bevacizumab monthly [17,18]	26.0 (69/265)	30.2 (39/129)		
	Bevacizumab PRN [17,18]	19.2 (52/271)	13.9 (35/251)		
	Aflibercept 2 mg q4 weeks [11,51]	64.8 (184/285)	44.6 (220/493)		
	Aflibercept 2 mg q8 weeks [11,51]	63.4 (168/265)	50.1 (253/505)		
**Adjusted mean ∆BCVA from baseline ^‡‡^** (total patients)	Faricimab (TENAYA trial) [12,13]	7.1 *	3.7 (337)		
	Aflibercept 2 mg (TENAYA trial) [12,13]	7.7 *	3.3 (334)		
	Faricimab (LUCERNE trial) [12,13]	-	5.0 (327)		
	Aflibercept 2 mg (LUCERNE trial) [12,13]	-	5.2 (331)		
**Adjusted mean ∆CST from baseline ^‡‡^** (total patients)	Faricimab (TENAYA trial) [12,13]	-	146.5 (337)		
	Aflibercept 2 mg (TENAYA trial) [12,13]	-	146.2 (334)		
	Faricimab (LUCERNE trial) [12,13]	-	150.3 (327)		
	Aflibercept 2 mg (LUCERNE trial) [12,13]	-	141.6 (331)		

^‡‡^ Patients were randomized 1:1 to faricimab 6.0 mg (up to q16 weeks after four initial monthly doses) or aflibercept 2 mg (q8 weeks after three initial monthly doses). Patients in the faricimab group were treated every 8, 12, or 16 weeks via protocol-defined disease activity criteria based on set anatomical BCVA measurements. After the first 60 weeks, the faricimab patients followed a (TAE) protocol until week 108, where dosing could be extended by 4 weeks (up to 16 weeks total) if the patient’s imaging demonstrated stable anatomy, vision was stable, and there were no signs of macular hemorrhage. If vision worsened, anatomy was not stable, or there was new macular hemorrhage then the interval was reduced by 4 or 8 weeks [12]. Furthermore, the first set of data is from the 48-week timepoint, thought to be representative of approximately one year of data. The second timepoint represent the averages of weeks 104–112, representative of approximately two years of data. * Japanese subgroup TENAYA analysis with up to q16 week dosing intervals.

## Data Availability

Supporting data can be found and is referenced within the publication. No other new data were created.

## Abbreviations

AMD	age-related macular degeneration
BCVA	best corrected visual acuity
CMT	central macular thickness
CST	central subfield thickness
EZ	Ellipsoid Zone
ETDRS	Early Treatment Diabetic Retinopathy Study
FDA	Food and Drug Administration
GA	geographic atrophy
ICER	incremental cost-effectiveness ratio
IRF	intra-retinal fluid
MNV	macular neovascularization
nAMD	neovascular age-related macular degeneration
OCT	optical coherence tomography
OD	oculus dexter (right eye)
OS	oculus sinister (left eye)
PlGF	Placental growth factor
PRN	pro re nata
QALY	quality-adjusted life year
RCT	randomized control trial
SRF	sub-retinal fluid
TAE	treat and extend
VA	visual acuity
VEGF	vascular endothelial growth factor
